# The mechano‐response of murine annulus fibrosus cells to cyclic tensile strain is frequency dependent

**DOI:** 10.1002/jsp2.1114

**Published:** 2020-07-20

**Authors:** Min Kyu M. Kim, Marissa J. Burns, Meaghan E. Serjeant, Cheryle A. Séguin

**Affiliations:** ^1^ Department of Physiology and Pharmacology Schulich School of Medicine & Dentistry, The University of Western Ontario London Ontario Canada; ^2^ Bone and Joint Institute, The University of Western Ontario London Ontario Canada

**Keywords:** annulus fibrosus, cyclic tensile strain, gene expression, intervertebral disk, mechanotransduction

## Abstract

The intervertebral disk (IVD) is a composite structure essential for spine stabilization, load bearing, and movement. Biomechanical factors are important contributors to the IVD microenvironment regulating joint homeostasis; however, the cell type‐specific effectors of mechanotransduction in the IVD are not fully understood. The current study aimed to determine the effects of cyclic tensile strain (CTS) on annulus fibrosus (AF) cells and identify mechano‐sensitive pathways. Using a cell‐type specific reporter mouse to differentiation NP and AF cells from the murine IVD, we characterized AF cells in dynamic culture exposed to CTS (6% strain) at specific frequencies (0.1 Hz, 1.0 Hz, or 2.0 Hz). We demonstrate that our culture model maintains the phenotype of primary AF cells and that the bioreactor system delivers uniform biaxial strain across the cell culture surface. We show that exposure of AF cells to CTS induces cytoskeleton reorganization resulting in stress fiber formation, with acute exposure to CTS at 2.0 Hz inducing a significant yet transient increase ERK1/2 pathway activation. Using SYBPR‐based qPCR to assess the expression of extracellular matrix (ECM) genes, ECM‐remodeling genes, candidate mechano‐sensitive genes, inflammatory cytokines and cell surface receptors, we demonstrated that exposure of AF cells to CTS at 0.1 Hz increased *Acan*, *Prg4*, *Col1a1* and *Mmp3* expression. AF cells exposed to CTS at 1.0 Hz showed a significant increase in the expression of *Acan*, *Myc*, and *Tnfα*. Exposure of AF cells to CTS at 2.0 Hz induced a significant increase in *Acan*, *Prg4*, *Cox2*, *Myc*, *Fos*, and *Tnfα* expression. Among the cell surface receptors assessed, AF cells exposed to CTS at 2.0 Hz showed a significant increase in *Itgβ1*, *Itgα5*, and *Trpv4* expression*.* Our findings demonstrate that the response of AF cells to CTS is frequency dependent and suggest that mechanical loading may directly contribute to matrix remodeling and the onset of local tissue inflammation in the murine IVD.

## 1 INTRODUCTION

According to the most recent *Global Burden of Disease Study*, back pain is the leading cause of years lived with disability worldwide.^1^ Although the etiology of chronic back pain remains largely unknown, persistent back pain has been associated with magnetic resonance imaging (MRI) findings of lumbar intervertebral disk (IVD) degeneration in ~30% of patients.^2^ The pathophysiology of IVD degeneration involves progressive cell‐mediated changes to the IVD microenvironment, including extracellular matrix (ECM) breakdown, altered matrix synthesis, and local tissue inflammation, ultimately resulting in structural and functional tissue failure.^3^, ^4^, ^5^, ^6^ Although the etiology of IVD degeneration is unclear, initiation and progression of the degenerative cascade involves multiple interdependent factors including altered mechanical loading,^7^, ^8^, ^9^, ^10^ reduced nutrient supply,^9^, ^10^, ^11^, ^12^ altered cellular composition,^3^, ^13^ and hereditary factors.^5^, ^14^, ^15^

The IVD is a fibrocartilaginous connective tissue structure essential for spine load bearing and movement. Anatomically, IVDs consist of three distinct tissues: the central gelatinous nucleus pulposus (NP), the outer collagenous annulus fibrosus (AF) that circumferentially encapsulates the NP, and the cartilage endplates that anchor the disk to the adjacent vertebrae and allow for passive diffusion of nutrients to the IVD.^8^, ^12^, ^16^, ^17^ As such, IVDs are heterogeneous composite structures with each tissue having a unique structure and specific ECM composition that together form the complex microarchitecture of the IVD required for joint function.^17^

Similar to other musculoskeletal tissues, mechanical loading poses an interesting dichotomy in IVD biology: while physiological levels of mechanical loading during moderate locomotive activities are essential for IVD health and tissue homeostasis,^10^, ^18^, ^19^ mechanical stimuli due to high and low degrees of physical activities (overloading and immobilization) can contribute to tissue degeneration.^18^, ^19^, ^20^ IVDs are subjected to various types of mechanical forces, including hydrostatic pressure, compressive, tensile and shear forces.^21^, ^22^, ^23^, ^24^ Within this dynamic microenvironment, NP cells are primarily exposed to compressive and hydrostatic loading, while AF cells are exposed to multidirectional deformation resulting in tensile strain.^21^, ^22^, ^23^, ^24^ Previous studies investigating the mechanical forces experienced by human IVDs reported that in performing daily activities, intradiscal pressures are predicted to range from 0.1 MPa (bedrest) to 2.5 MPa (heavy lifting).^25^ Furthermore, studies characterizing the mechanical properties and strain profiling of human IVDs reported that the AF experiences 1% to 13% strain during daily activities,^26^ with tissue strain reaching up to 26% during physiological compressive loading.^27^

To better understand how the dynamic environment of the IVD regulates cell function, studies have examined the effects of cyclic tensile strain (CTS) on AF cells to model their exposure to tensile loading in vivo. In response to uniaxial CTS (2%, 1.0 Hz), human AF cells in 3D culture show increased aggrecan gene expression and decreased expression of matrix degrading enzymes.^28^ Using the same model system, exposure of AF cells to 4% CTS (1.0 Hz) induced a modest increase in matrix gene expression, dependent on osmotic potentials.^29^ In contrast, exposure of human AF cells to high levels of bi‐axial strain (20% CTS, 0.001 Hz) induced catabolic responses, including upregulation of inflammatory cytokine production and cell death.^30^ Studies using the Flexcell tension system reported that human AF cells exposed to 10% CTS at 1.0 Hz showed increased expression of matrix genes and decreased expression of matrix degrading enzymes, whereas 10% CTS frequency at 0.33 Hz shifted the mechano‐response toward matrix catabolism.^31^, ^32^ Of note, application of either loading protocol to human AF cells from degenerate IVDs increased catabolic gene expression.^30^

To date, reports on the mechano‐sensory mechanism of AF cells have focused on the role of integrin‐ECM interactions in CTS sensing^32^, ^33^; however, specific signaling pathways involved in mechanotransduction remain largely unexplored. Mitogen‐activated protein kinases (MAPKs) are a family of highly conserved protein kinases that serves as intermediates in signal transduction pathways.^34^ MAPKs are grouped into three subfamilies—extracellular signal‐regulated kinases (ERKs), p38, and c‐jun N‐terminal or stress‐activated kinases (JNK/SAPK)—based on their sequence, sensitivity to activation and mechanisms of action.^35^ Functionally, ERKs regulate cell survival and proliferation,^36^ whereas p38 kinases regulate inflammatory responses, cell cycle control, and differentiation.^37^ Interestingly, AF cells isolated from degenerative human IVDs show activation of all three MAPK subfamilies (ERK1/2, p38, JNK) upon mechanical stimulation.^38^

The current study used a transgenic mouse model to genetically label and isolate AF cells from the murine IVD and employed an in vitro culture system to deliver acute bi‐axial CTS. Bi‐axial multidirectional load was chosen to model complex circumferential tensile load experienced by the AF cells in vivo when the tissue is subjected to multiaxial load constrained by the adjacent vertebral bodies (axial and radial load).^23^, ^27^ The study aimed to quantify the mechano‐response of healthy murine AF cells to different loading conditions, focusing on cytoskeletal adaptation, changes in gene expression, and the identification of CTS‐induced signaling pathways.

## 2 MATERIALS AND METHODS

### 2.1 Animals

To differentiate cell types within the IVD, the notochord‐specific Noto^*Cre*^ mouse strain reported by our group^39^ was mated to the conditional *ROSA26* (*R26*) *mT/mG* reporter mouse (*Gt*(*ROSA*)*26Sor* ^*tm4(ACTB‐tdTomato*,*‐EGFP)Luo*^/J).^40^ Genotyping was performed as previously described.^39^ IVD tissues from *Noto* ^*Cre/WT*^;*Rosa* ^*mTmG/mTmG*^ mice, in which notochord‐derived NP cells are marked by green fluorescent protein (GFP) and AF cells express tdTomato, were used for all experiments. Mice were housed in standard cages and maintained on a 12‐hours light/dark cycle, with rodent chow and water available ad libitum. Mice were euthanized by CO_2_ asphyxiation at 2 months‐of‐age for tissue isolation. All animal experiments were performed in accordance with the policies and guidelines set forth by the Canadian Council on Animal Care and were approved by the Animal Use Subcommittee of the University of Western Ontario (protocol 2017‐154).

### 2.2 Tissue isolation and culture of AF cells

**FIGURE 1:**
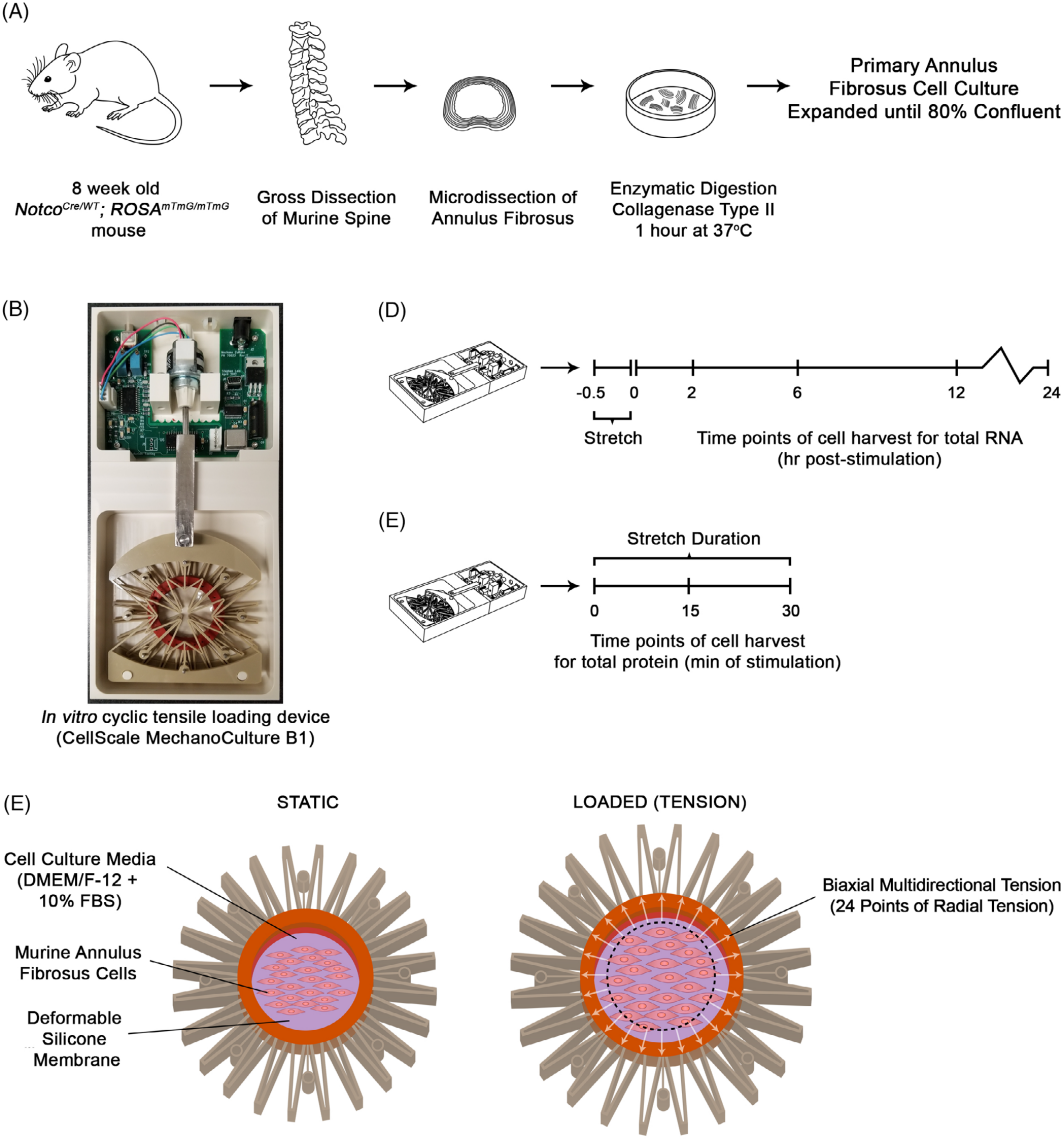
Experimental design and mechanical stimulation of murine annulus fibrosus cells. A, Schematic representation of the protocol used for the isolation and culture of primary murine annulus fibrosus cells. B, Images of the MechanoCulture B1 device inside the cell culture incubator. C, Schematic depicting the in vitro model used to deliver cyclic tensile strain. Cells were seeded on a deformable silicone membrane puncture mounted on a 24‐pin mounting ring designed to generate radial motion from a linear motion input, resulting in the delivery of biaxial stretch. D, E, Schematic representations of the experimental procedures used to deliver 6% cyclic tensile strain at frequencies of 0.1, 1.0, or 2.0 Hz to investigate gene expression, C, and signal transduction, D, in mechanically stimulated annulus fibrosus cells

Lumbar spines from 2‐month‐old mice were dissected, followed by microdissection of NP and AF tissues using a fluorescent stereo microscope (Leica M165 FC). Isolated tissues were immediately fixed for RNA extractions. For primary cell culture (overview in Figure 1A), intact IVDs were dissected from 2‐month‐old mice (cervical to caudal) and the AF tissues were microdissected (Leica M165 FC). Isolated AF tissues were transferred to a sterile 3 mm culture dish with 2 mL of type II collagenase (3 mg/mL; Worthington, NJ) in Dulbecco's modified Eagle's medium/Ham's F‐12 medium (DMEM/F12) and incubated for 20 minutes at 37°C. AF tissues were then minced and further digested for 1 hour at 37°C. Digested tissues were triturated and filtered using a 70 μm cell strainer and cells were pelleted by centrifugation (1100 rpm for 5 minutes). Cells were plated at an initial density of ~400  000 cells/cm^2^ and cultured in DMEM/F12 supplemented with 10% fetal bovine serum (FBS) and 1% penicillin and streptomycin (Thermo Fisher Scientific, MA) at 37°C in a humidified atmosphere of 5% CO_2_. Media was changed every 2 days until cells reached 80% confluency. AF cells isolated from IVD tissues of two mice were pooled together and used for each experimental replicate.

### 2.3 Mechanical stimulation

The MechanoCulture B1 (MCB1) device (CellScale Biomaterials Testing, Waterloo, Ontario, Canada) was used to deliver bi‐axial multidirectional cyclic tensile strain to monolayer cultures (Figure 1B,C).^41^ The MCB1 device included an actuator chamber, housing a programmable circuit board and motor (Figure 1B, top), connected by a stainless steel arm to a loading chamber containing the deformable construct on which the cells were grown (Figure 1B, bottom). Three deformable polyether ether ketone plastic layers were assembled in order to transfer the linear motion of the steel arm into a radial stretch of an inner circle (35 mm diameter) of 24 pins. Clear silicone membranes (0.005″ thickness, ultimate tensile strength 8.62 MPa, McMaster‐Carr, Aurora; #87315 K71) were puncture‐mounted on the device's 24‐pin mounting ring and the assembled constructs were sterilized by standard gravity displacement steam autoclave cycle (30 minutes sterilization, 20 minutes cool; temperature during sterilization = 121.1°C‐123.3°C). Sterilized silicone membranes were then coated with 2 mL of 50% FBS DMEME/F12 overnight. Primary AF cells were seeded at a density of 48  000 cells/cm^2^ onto the FBS‐coated silicone membrane and cultured for 2 days in culture media supplemented with 50 ng/μL l‐ascorbic acid. Once monolayers reached 80% confluency, the assemblies were transferred to the loading chamber of the device within the culture incubator. AF cells were exposed to 6% CTS, at sinusoidal frequencies of either 0.1 Hz, 1.0 Hz, or 2.0 Hz for 30 minutes. AF cells cultured on FBS‐coated silicone membranes without mechanical stimulation served as time‐matched unloaded controls. After 30 minutes of CTS, cells were incubated for additional 2, 6, 12, or 24 hours before harvesting for total RNA, with time‐matched unloaded cells serving as control.

### 2.4 Motion tracking analysis

To quantify the magnitude and uniformity of the strain applied to the silicone membrane by the MCB1 device, motion tracking analysis was performed. Fine graphite powder was used to pattern the silicone membrane in order to quantify the motion of specific points during mechanical stretch. The patterned surface was imaged at 0.5 mm intervals using DMK 41AU02 Monochrome Camera (Imaging Source, Charlotte, NC) until the programmed displacement (3.5 mm) was reached. Using commercially available image tracking software (LabJoy, CellScale), four different regions of the membrane were tracked and the strains experienced in these regions were calculated based on pixel displacement.

### 2.5 Immunofluorescence analysis

Immediately following mechanical stimulation, AF cells on the silicone membranes were fixed with 4% paraformaldehyde for 10 minutes and then permeabilized with 0.1% Triton X‐100 (in phosphate buffered saline [PBS]) for 10 minutes at room temperature. After blocking in 1% bovine serum albumin (in PBS) for 30 minutes, Alexa Fluor 488 Phalloidin (Life Technologies, Carlsbad, CA) was used to detect F‐actin according to manufacturer's protocol and the nuclei detected using Hoeschst stain (Thermo Fisher). Images were acquired using a Zeiss Axio Observer 7 with AxioVision software (Carl Zeiss, Jena, Germany). In each of the four biological replicates, cells within three nonoverlapping regions of interest (ROIs) were imaged for each experimental group (unloaded, 0.1 Hz, 1.0 Hz, and 2.0 Hz) for a total of 12 images for each experimental group. Each image was analyzed by counting total number of cells (5‐9 cells per ROI) and categorizing each cell as either stress fiber‐negative (cells with intense F‐actin staining localized near cell periphery with either punctate or weak fiver staining in cytoplasm) or stress fiber‐positive (condensed F‐actin fiber staining). For each experimental group, total of 63 to 84 cells were examined. Cells staining positive for stress fibers are presented as percentage of total cells counted for each experimental group.

### 2.6 Protein isolation and Western blot analysis

AF cells were cultured on the silicone membranes in the MCB1 device as described above and transferred to serum‐free DMEM/F12 media 24 hours prior to loading. AF cells were subjected to 6% CTS at frequencies of either 1.0 Hz, 2.0 Hz, for either 15 or 30 minutes (n = 3). Immediately following mechanical loading, AF cells were scraped from silicone membranes and lysed using RIPA buffer containing protease inhibitor (cOmplete Mini, EDTA‐free Protease Inhibitor Cocktail, Sigma‐Aldrich Canada). Samples were centrifuged at 14 000*g* for 15 minutes at 4°C. The supernatant was collected, and protein concentration was determined using the BCA Protein Assay Kit (Pierce Biotechnology, Rockford, IL) according to the manufacturer's protocol. For each sample, 21 μg of protein was loaded and resolved by sodium dodecyl sulfate polyacrylamide gel electrophoresis (10% gels) and proteins were transferred to Immobilon Polyvinylidene fluoride membranes (Sigma‐Aldrich) using the Trans‐Blot Turbo Blotting System (Bio‐Rad). Membranes were blocked with 5% w/v nonfat dried milk in tris‐buffered saline‐tween (TBST) and incubated with primary antibody against Phospho‐p38 MAPK Thr180/Tyr182 (Cell Signaling Technology, #4511, 1:1000), p38 MAPK (Cell Signaling Technology, #8690, 1:1000), Phospho‐p44/42 MAPK Erk1/2 Thr202/Tyr204 (Cell Signaling Technology, #4370, 1:1000), or p44/42 MAPK Erk1/2 (Cell Signaling Technology, #4370, 1:1000) in TBST with 5% milk (Cell Signaling Technology, Danvers, MA). After washing with TBST, membranes were incubated with anti‐rabbit horseradish peroxidase‐linked secondary antibody (Santa Cruz Biotechnology, Dallas, TX, sc‐2004; 1:2000). Protein bands were visualized by chemiluminescence using the enhanced chemiluminescence kit (Bio‐Rad, Hercules, CA) and imaged using the ChemiDOC XRS + System (Bio‐Rad). Densitometric analysis was performed using ImageLab software (Bio‐Rad). Levels of phospho‐proteins were quantified and presented normalized to corresponding total protein levels.

### 2.7 RNA extraction and gene expression analysis

Total RNA was extracted from IVD tissues, primary AF cells cultured on standard tissue culture plastic or silicone membrane (passage 1) or CTS‐treated and unloaded AF cells 2, 6, 12, or 24 hours post‐stimulation (n = 4‐7) using Trizol reagent (Life Technologies) according to the manufacturer's protocol. RNA was quantified using a NanoDrop 2000 spectrophotometer (Thermo Fisher Scientific). Complementary DNA was synthesized from 150 ng of RNA using the Bio‐Rad iScript cDNA synthesis kit. Gene expression was determined by SYBR‐based real‐time PCR using the Bio‐Rad CFX384 thermocycler. PCR reactions were run in triplicate using 470 nM forward and reverse primers (primer sequences in Table 1) with 2x SsoFast EvaGreen Supermix (Bio‐Rad). The PCR program consisted of the following: initial 2 minutes enzyme activation at 95°C, 10 seconds denaturation at 95°C, 30 seconds annealing/elongation at 60°C, for total of 40 cycles. For cell phenotype characterization, transcript levels were determined relative to a six‐point calibration standard curve made from pooled cDNA generated from wild type murine heart, brain, kidney, muscle, IVD, and mouse embryonic fibroblasts. For CTS experiments, gene expression values were calculated using ∆∆Ct, normalized for input based on hypoxanthine quinine phosphoribosyl transferase (*Hprt*) expression and expressed relative to the time‐matched unloaded controls within each trial.

**TABLE 1:** Sequences of the primers used in the real‐time PCR analysis

Gene	Forward (5′ ➔ 3′)	Reverse (5′ ➔ 3′)
*Acan*	CCTGCTACTTCATCGACCCC	AGATGCTGTTGACTCGAACCT
*Acta2*	GTCCCAGACATCAGGGAGTAA	TCGGATACTTCAGCGTCAGGA
*Adamts4*	GAGGAGGAGATCGTGTTTCCAG	CAAACCCTCTACCTGCACCC
*Bgn*	ACGAATCCATGACAACCGTATC	GCTCCTGGTTCAAAGCCACT
*Cd24*	ACCCACGCAGATTTACTGCAA	CCCCTCTGGTGGTAGCGTTA
*Cilp*	ATGGCAGCAATCAAGACTTGG	AGGCTGGACTCTTCTCACTGA
*Col1a1*	CTGGCGGTTCAGGTCCAAT	TCCAGGCAATCCAGGAGC
*Col2a1*	GCACATCTGGTTTGGAGAGACC	TAGCGGTGTTGGGAGCCA
*Col10a1*	GGGACCCCAAGGACCTAAAG	GCCCAACTAGACCTATCTCACCT
*Cox2*	GGCGCAGTTTATGTTGTCTGT	CAAGACAGATCATAAGCGAGGA
*Dcn*	TCTTGGGCTGGACCATTTGAA	CATCGGTAGGGGCACATAGA
*Fap*	GTCACCTGATCGGCAATTTGT	CCCCATTCTGAAGGTCGTAGAT
*Fos*	CGGGTTTCAACGCCGACTA	TTGGCACTAGAGACGGACAGA
*Gdf10*	GAAGTACAACCGAAGAGGTGC	AGGCTTTTGGTCGATCATTTCC
*Hprt*	CAGGCCAGACTTTGTTGGAT	TTGCGCTCATCTTAGGCTTT
*Il‐1β*	CCCTGCAGCTGGAGAGTGTGGA	TGTGCTCTGCTTGTGAGGTGCTG
*Il‐6*	TCTCTGCAAGAGACTTCCATCCAGT	AGTAGGGAAGGCCGTGGTTGTCA
*Itgβ1*	ACTGATTGGCTGGAGGAATGTTAC	CTGGACAAGGTGAGCAATAGAAGG
*Itgα5*	CTTCTCCGTGGAGTTTTACCG	GCTGTCAAATTGAATGGTGGTG
*Mmp3*	TTGTCCCGTTTCCATCTCTCTC	TTGGTGATGTCTCAGGTTCCAG
*P2rx7*	GGCACTGGAGGAAAATTTGA	TGAGCAAGTCAATGCACACA
*Prg4*	GGGTGGAAAATACTTCCCGTC	CAGGACAGCACTCCATGTAGT
*Timp1*	CTTGGTTCCCTGGCGTACTC	ACCTGATCCGTCCACAAACAG
*Tlr2*	CACCACTGCCCGTAGATGAAG	AGGGTACAGTCGTCGAACTCT
*Tlr4*	GCCTTTCAGGGAATTAAGCTCC	GATCAACCGATGGACGTGTAAA
*Tnfα*	TCGGGGTGATCGGTCCCCAA	GGTGGTTTGCTACGACGTGGGC
*Trpv4*	TTCGTAGGGATCGTTGGTCCT	TACAGTGGGGCATCGTCCGT

### 2.8 Statistical Analysis

All statistical analyses were performed with GraphPad Prism 6 Software (GraphPad Software, San Diego, CA). The analysis of data from stress fiber quantification and gene expression for cell phenotype characterization were analyzed using one‐way analysis of variation (ANOVA) followed by Tukey's multiple comparison test. Western blot data were analyzed using Krustkal‐Wallis and Dunn's post hoc test. For CTS experiments, gene expression levels in AF cells exposed to CTS at varying frequencies were compared to unloaded controls at each time point using one‐way ANOVA with Dunnett's post hoc test. For each gene, differences in expression values between CTS‐treated AF cells harvested at specific time points poststimulation were compared using one‐way ANOVA with Tukey's post hoc test. In order to test whether frequency is a source of variation for a given gene, differences in gene expression values between different CTS protocols at each time point were analyzed using two‐way ANOVA followed by Tukey's post hoc test. *P* values less than .05 were considered statistically significant.

## 3 RESULTS

### 3.1 MechanoCulture B1 device delivers uniform cyclic tensile loading

**FIGURE 2:**
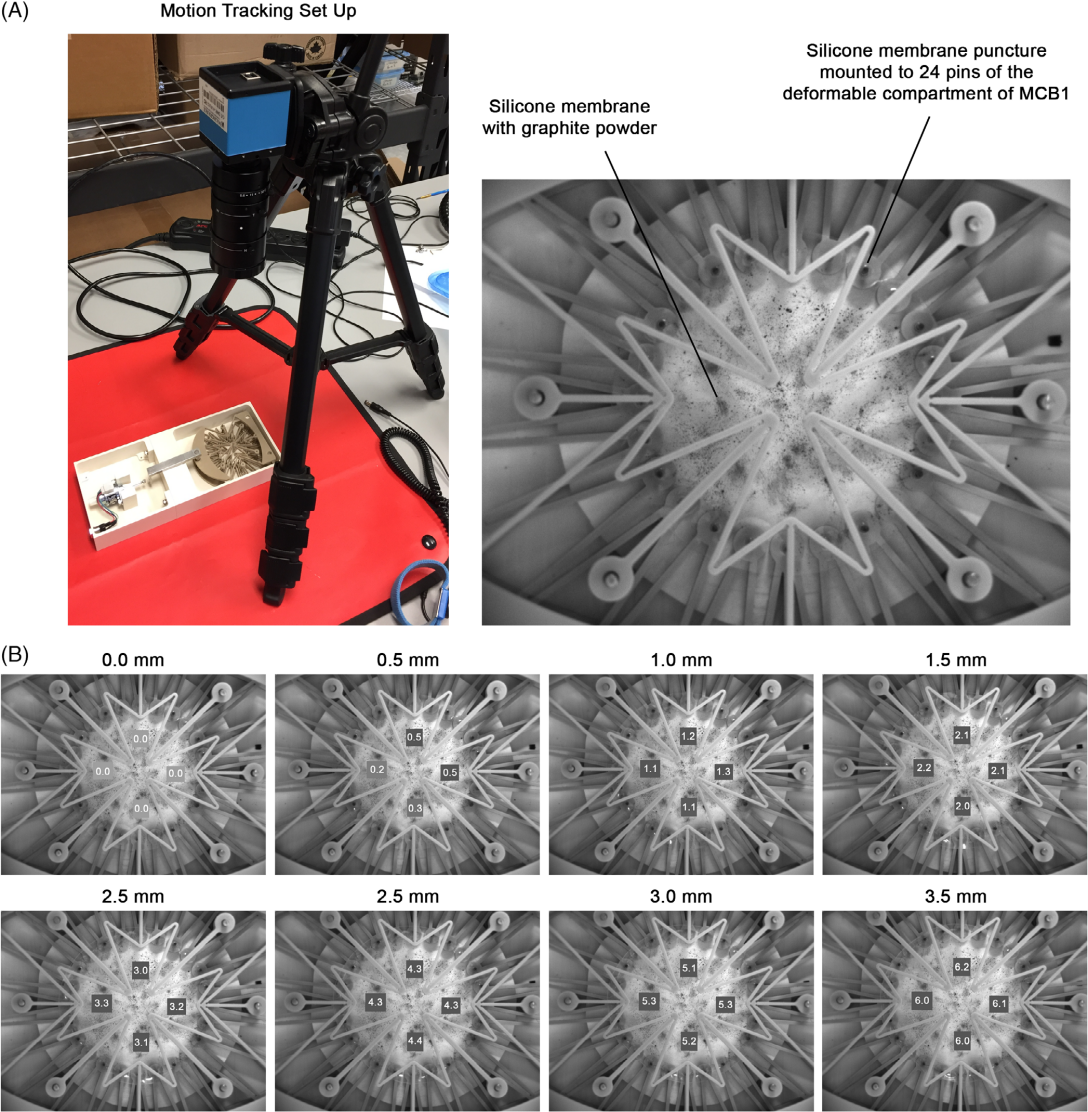
Motion tracking analysis of the MechanoCulture B1 device to validate strain profile. A, Silicone membrane was textured using graphite powder and the membrane was stretched and imaged every 0.5 mm until the programmed total displacement (3.5 mm) was reached. B, Four different regions of interest (ROI) on the membrane were tracked and strains experienced in these regions were calculated based on pixel displacement (values indicate within ROIs at each displacement). The analysis demonstrated that a 3.5 mm displacement produced uniform 6% on the silicone cell culture membrane

To quantify the magnitude and uniformity of the strain applied to the silicone culture membrane, motion tracking analysis was performed. The device was programmed for a 3.5 mm linear displacement, a load translated into 24 points of radial motion by the device (Figure 1B, C). Regions of interest (ROIs) on the silicone membrane were tracked and imaged every 0.5 mm during the displacement and the strain calculated based on pixel displacement (Figure 2A). This analysis demonstrated that the average strain experienced by the ROIs on the silicone membrane at full displacement (3.5 mm) was 6.075 ± 0.096%. The small variability in strain measured between ROIs suggests that strain across the membrane at full displacement is uniform (Figure 2B).

Previous studies predicted that the AF experiences compressive tissue strains of 1‐26%, radial tensile strain of 1‐19%, and lamellar fiber strain of up to 13% when IVD is compressed with a load physiologically similar to that of walking.^26^, ^27^, ^42^ Given such data, CTS of 6% was chosen as it falls within the physiological range of mechanical stimulation in vivo. Frequency of 1.0 Hz was chosen as being representative of physiologic locomotion, and frequencies of 0.1 Hz and 2.0 Hz were chosen as being less than and greater than physiologic locomotion, respectively^43^ (0.1 Hz = bed rest, 1.0 Hz = walking gait, 2.0 Hz = steady running gait).

### 3.2 Primary murine annulus fibrosus cells maintain an AF‐like phenotype in culture

**FIGURE 3:**
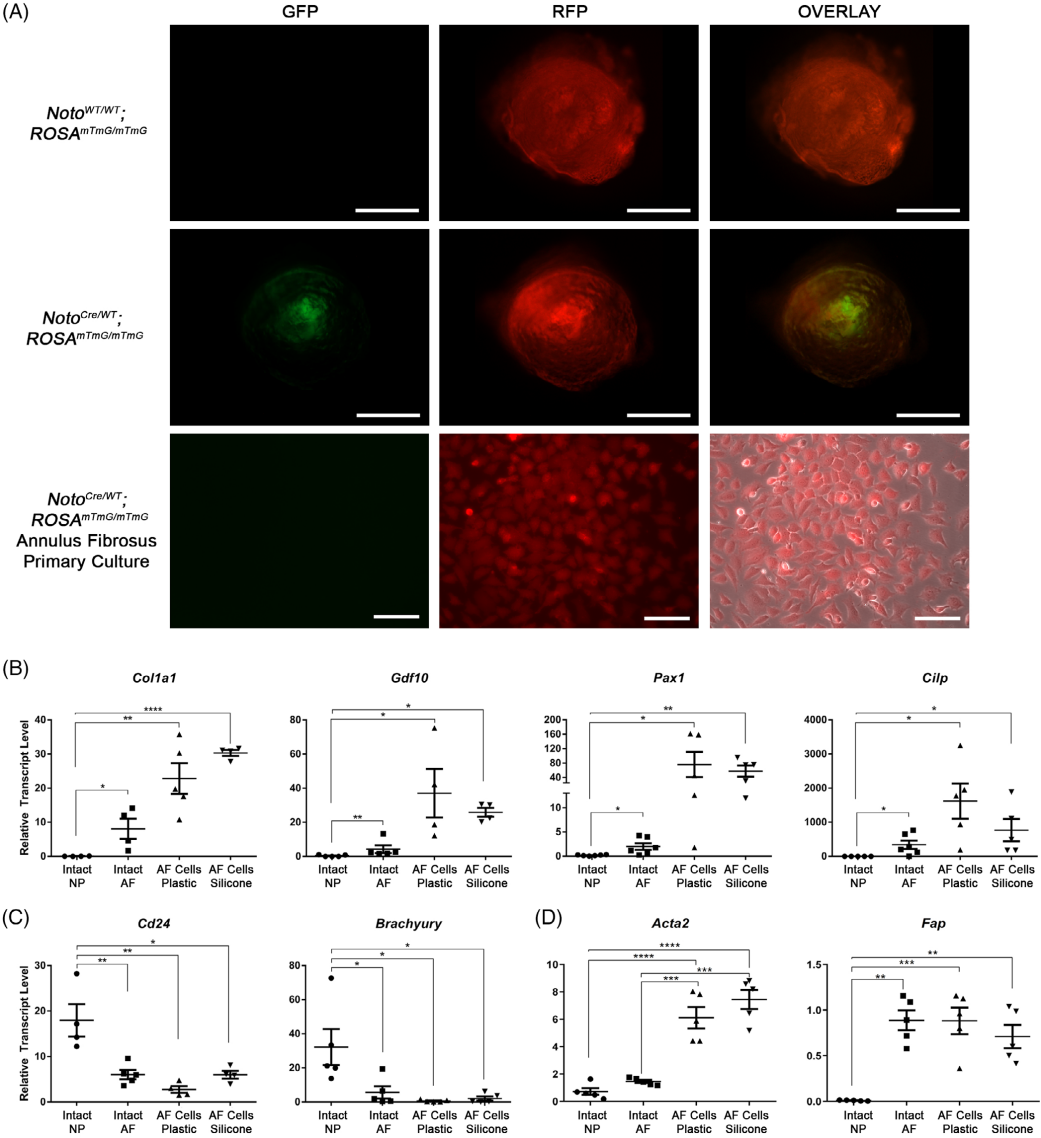
Analysis of cell phenotype in primary annulus fibrosus (AF) cell cultures. Primary AF cells were isolated from the *Noto* ^*Cre/WT*^; *ROSA* ^*mTmG/mTmG*^ conditional reporter mouse to allow for cell isolation based on fluorescence. A, Fluorescent micrograph of intact IVD isolated from *Noto* ^*WT/WT*^; *ROSA* ^*mTmG/mTmG*^ and *Noto* ^*Cre/WT*^; *ROSA* ^*mTmG/mTmG*^ mice showing NP‐specific CRE activity. AF cells isolated from the conditional reporter show RFP but not GFP expression, indicating the absence of NP cell contamination. B‐D, Gene expression analysis of primary annulus fibrosus cells compared to intact IVD tissues using panels of AF‐ and NP‐associated markers and fibroblast markers. The expression of selected genes was quantified using qRT‐PCR in primary AF cells grown on standard tissue culture plastic or silicone membranes (n = 5) and compared to intact AF and NP tissues (n = 6 mice; 5‐6 IVDs pooled per mouse). B, AF cells cultured on tissue culture plastic and silicone membranes maintained expression of the AF markers, *Col1a1*, *Gdf10*, *Pax1*, and *Cilp* at levels comparable to intact AF tissue, significantly higher compared to NP tissue. C, AF cells cultured on tissue culture plastic and silicone membranes exhibited significantly lower expression of the NP markers, *Cd24* and *Brachyury* compared to NP tissues. D, AF cells cultured on tissue culture plastic and silicone membranes had significantly increased expression of *Acta2* compared to both intact IVD tissues; however, *Fap* expression was not significantly altered in AF cell cultures compared to intact AF tissue. Grubb's outlier test was used to identify outliers. Data presented as mean ± SEM. Data analyzed using one‐way analysis of variation (ANOVA) followed by Tukey's post hoc test. *P < .05; **P < .01, ***P < .001; ****P < .0001. Scale bars = tissue micrograph = 200 μm; cell culture = 100 μm

To allow cell type‐specific isolation from the murine IVD, primary cells were isolated from the *Noto* ^*cre*^;*ROSA* ^*mTmG/mTmG*^ conditional reporter mice in which AF cells express red fluorescent protein (RFP), whereas notochord‐derived NP cells express GFP (Figure 3A). In all AF cell preparations maintained in monolayer culture, only RFP‐expressing cells were observed, confirming the absence of notochord‐derived NP cells (Figure 3A).

To ensure that primary AF cells maintained their phenotype in our in vitro culture system, we quantified the expression of previously reported AF‐ and NP‐associated markers^44^, ^45^, ^46^, ^47^, ^48^, ^49^, ^50^ as well as fibroblast markers and compared expression levels to those of the intact AF and NP tissues. Primary AF cells showed robust expression of the AF‐associated markers, type I collagen (*Col1a1*), growth differentiation factor (*Gdf10*), paired box 1 (*Pax1*) and cartilage intermediate layer protein (*Cilp*), compared to expression of these genes in the NP tissues (Figure 3B). No significant differences were detected in the expression of AF markers in primary cells cultured on tissue culture plastic or silicone membranes compared to the intact AF tissues. In contrast, AF cells showed minimal or no detectable expression of the NP‐associated markers, *Cd24* and *Brachyury*, with no differences detected between AF tissue and primary cells cultured on either tissue culture plastic or silicone membranes (Figure 3C). Moreover, we quantified the expression of the markers of the myofibroblast/activated fibroblast phenotype, alpha smooth muscle actin (*Acta2*),^51^ and fibroblast activation protein (*Fap*).^52^ Primary AF cells cultured on tissue culture plastic and silicone membranes showed significantly increased expression of *Acta2* compared to intact AF and NP tissues; however, expression of *Fap* was not altered in cultured AF cells compared to intact AF tissues (Figure 3D).

### 3.3 Mechanical stimulation induces cytoskeletal rearrangement in annulus fibrosus cells

**FIGURE 4:**
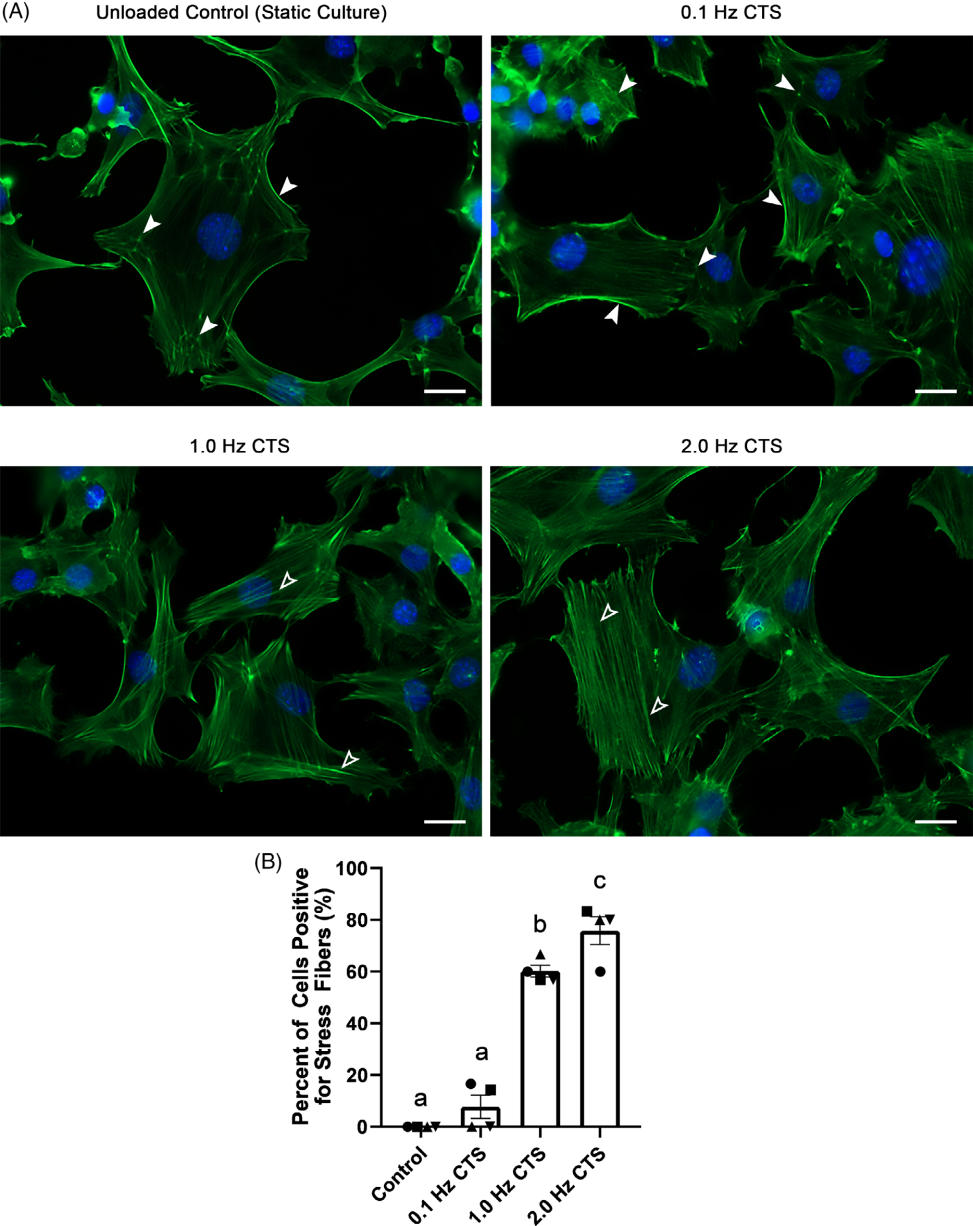
Cytoskeletal rearrangement in annulus fibrosus (AF) cells exposed to cyclic tensile strain. A. Representative images of AF cells in which F‐actin was visualized with alexa 488 phalloidin (counter stained with Hoechst stain to visualize nuclei) in AF cells grown in static culture (unloaded control) or following 30 minutes exposure to cyclic strain (CTS) at 0.1, 1.0, or 2.0 Hz. In the unloaded control and 0.1 Hz, F‐Actin was predominantly localized near the cell periphery with punctate distribution or weak fiber staining in the cytoplasm (white solid arrow). In contrast, AF cells exposed to CTS at 1.0 Hz and 2.0 Hz showed distinct stress fiber formations (hollow arrow). B, For each treatment group, the percentage of stress fiber‐positive cells was assessed. Graphs present the average percentage of stress fiber‐positive cells (calculated from three independent regions of interest) for each treatment group, for each of the four biological replicate experiments (presented as matching symbol). Compared to the unloaded control, exposure of AF cells to CTS at 1.0 or 2.0 Hz induced a significant increase in number of stress fiber‐positive cells. Data presented as mean ± SEM from four independent experiments. Bars labeled with the same letter are not significantly different based on *P* < .05; one‐way analysis of variation (ANOVA) followed by Tukey's post hoc test.; Scale bar = 20 μm

To confirm that AF cells sense and respond to the CTS delivered by the MCB1 device, cytoskeletal rearrangement was examined since cytoskeletal reorganization has been shown to play a pivotal role in AF mechanotransduction.^53^ In AF cells under static culture (unloaded control), F‐actin was localized near the cell periphery in a predominantly punctate distribution with weak fiber staining. This pattern of F‐actin staining was similar following 30 minutes exposure of cells to CTS at 0.1 Hz (Figure 4A solid arrow); however following exposure to CTS at 1.0 and 2.0 Hz, AF cells showed significantly increased stress fiber formation, which increased with higher frequency of loading (Figure 4A hollow arrow). Upon quantification, 61% of cells and 74% of cells were positive for stress fibers in AF cells exposed to CTS at 1.0 Hz and 2.0 Hz protocol, respectively (Figure 4B). The stress fibers formed in AF cells exposed to CTS at 1.0 and 2.0 Hz showed a random orientation.

### 3.4 Cyclic tensile strain activates ERK1/2 signaling in AF cells

**FIGURE 5:**
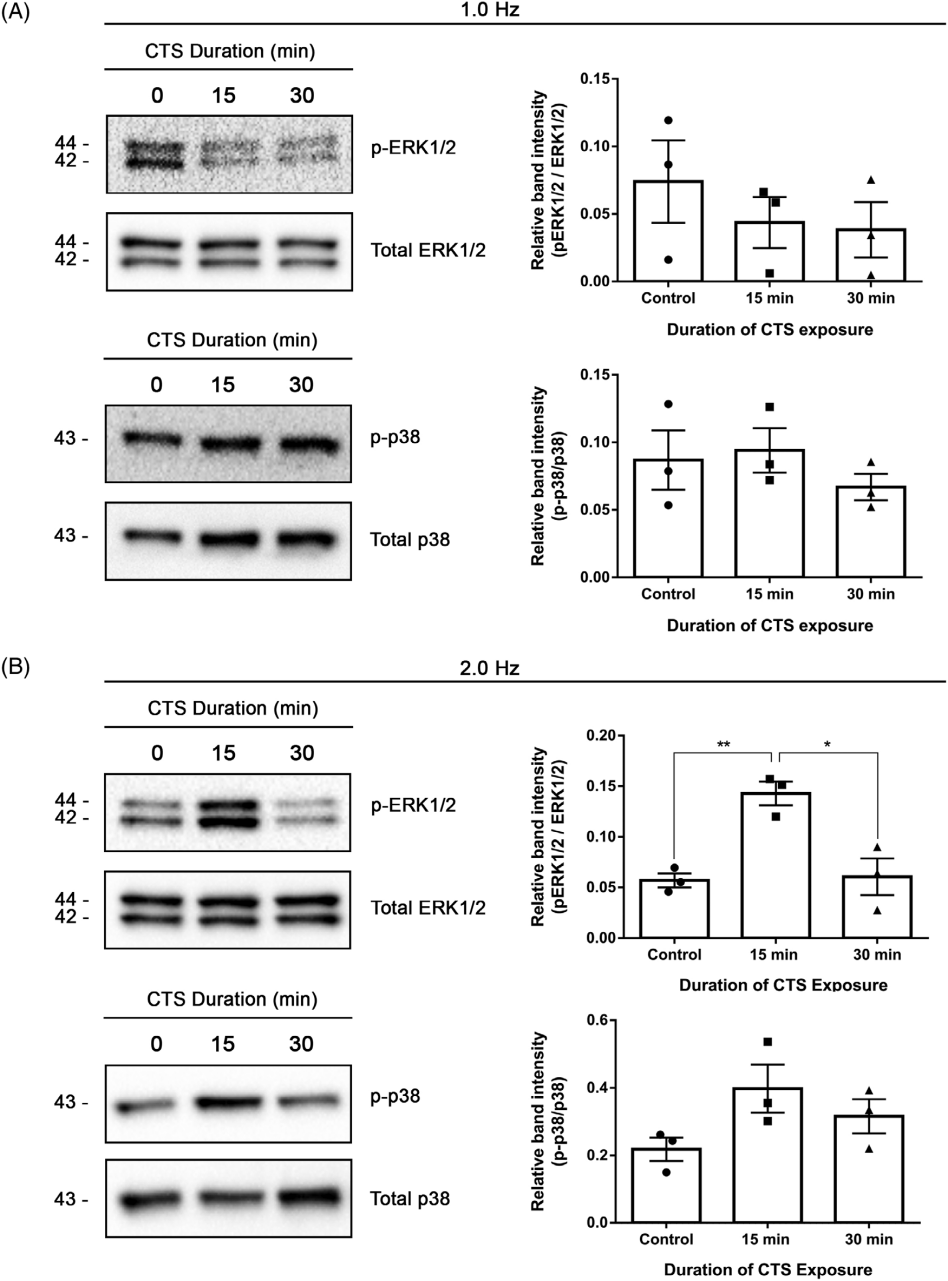
ERK1/2 and p38 pathway activation in annulus fibrosus (AF) cells following cyclic tensile strain. AF cells were subjected to 6% CTS for 15 or 30 minutes at frequencies of either 1.0 Hz, A, or 2.0 Hz, B. Cells cultured on silicone membranes in the absence of mechanical loading served as a control (0 CTS column). Total protein was harvested from AF cells immediately after loading and MAPK pathway activation was assessed through immunoblotting using antibodies for the phosphorylated forms (upper panels) or total (lower panels) ERK1/2 and p38 (bands representative of n = 3 cell preparations, 2 animals per cell preparations). Quantification was performed by measurement of average relative density of bands corresponding to phosphoproteins, normalized to total protein. Data presented as a mean SEM; data were analyzed using Kruskal‐Wallis test and Dunn's post hoc test. *P < .05; **P < .01

After validating our model of dynamic cell culture, we first assessed the effects of CTS on MAPK pathway activation. Exposure of AF cells to 15 or 30 minutes CTS at 1.0 Hz did not induce a significant activation of either the ERK1/2 or p38 MAPK pathways, compared to static (unloaded) control (Figure 5A). Exposure of AF cells to CTS at 2.0 Hz induced a transient increase in ERK1/2 phosphorylation, significantly increased following 15 minutes of CTS but not significantly different from static control following 30 minutes CTS (Figure 5B). A similar trend was detected for p38 activation; however, changes in levels of phosphorylation did not reach significance (Figure 5B).

### 3.5 Acute exposure of AF cells to CTS induces frequency‐dependent changes in gene expression

Since the effects of CTS on AF cell cytoskeletal rearrangement and MAPK pathway activation varied based on the frequency of load applied, we aimed to characterize the corresponding changes in gene expression using real‐time PCR, focusing on expression of ECM genes, matrix degrading enzymes, inflammatory cytokines, candidate mechanosensitive genes, and cell surface receptors identified in related musculoskeletal cell types.^9^, ^30^, ^32^, ^54^, ^55^, ^56^, ^57^, ^58^, ^59^, ^60^, ^61^ Given differences in the dynamic regulation of gene expression, cells were exposed to a single protocol of CTS (6% strain at 0.1, 1.0, or 2.0 Hz) and RNA was harvested following 2, 6, 12, or 24 hours post stimulation.

**FIGURE 6:**
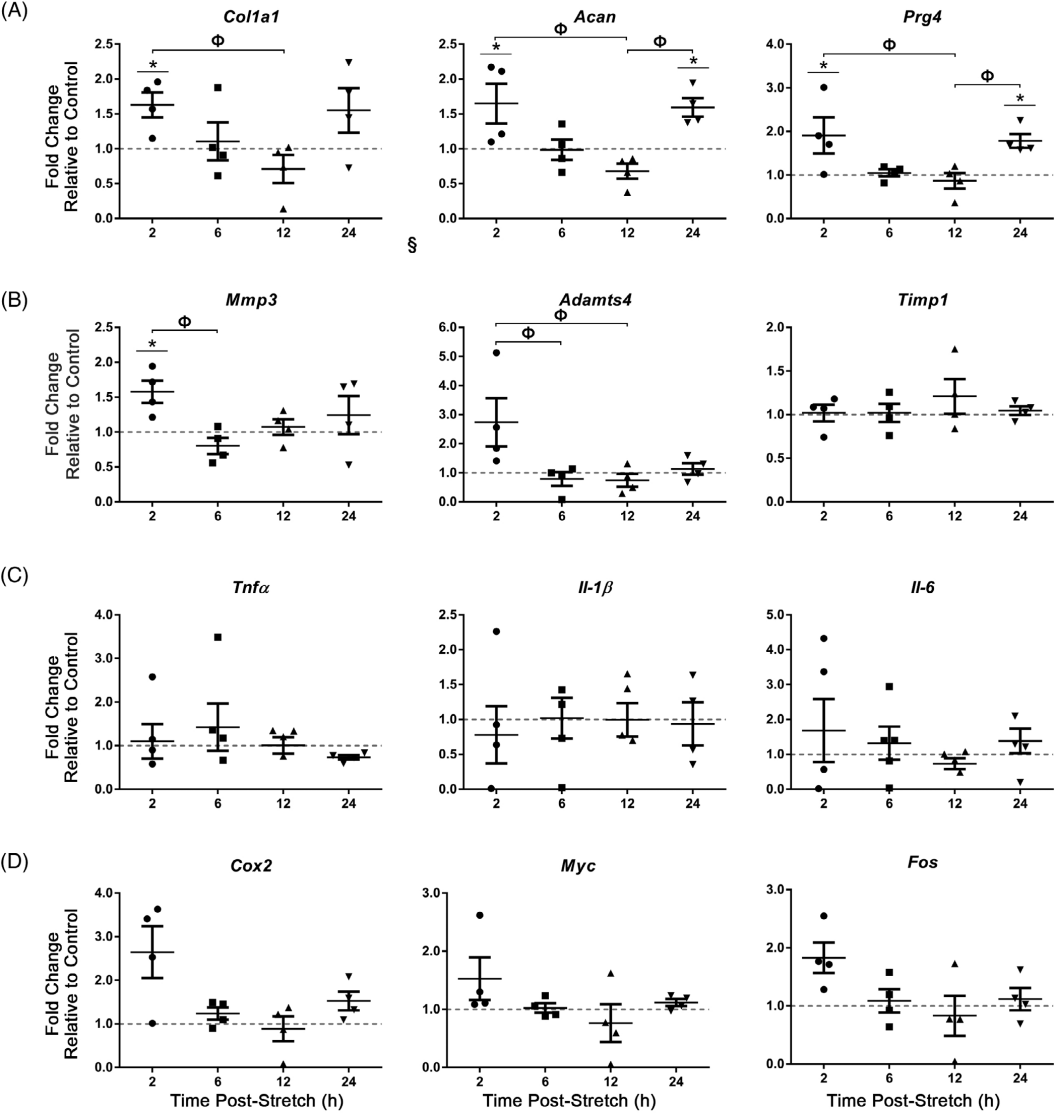
Real‐time PCR analysis of gene expression in annulus fibrosus (AF) cells following acute exposure to CTS at 0.1 Hz. Primary AF cells were subjected to 6% CTS at 0.1 Hz for 30 minutes and RNA harvested following 2, 6, 12 or 24 hours to assess the expression of extracellular matrix genes, A, matrix remodeling genes, B, inflammatory cytokines, C, and candidate mechanosensitive genes, D. AF cells showed a significant increase in the expression of *Col1A1* (2 hours postloading), *Prg4* (2 hours, 24 hours postloading), *Acan* (2 hours, 24 hours postloading) and *Mmp3* (2 hours postloading). Relative gene expression was calculated using ∆∆Ct, normalized for input using the housekeeping gene *Hprt* and expressed relative to time‐matched unloaded controls within each trial (control = 1; indicated as gray dotted lines). Data presented in mean ± SEM; n = 4 cell preparations. Data were analyzed using one‐way analysis of variation (ANOVA) followed by either Dunnett's or Tukey's post hoc test. Grubb's outlier test used to identify outliers. *P < .05 vs unloaded control; Ф = *P* < .05 between fold changes at two time points

AF cells exposed to acute (30 minutes) 6% CTS at 0.1 Hz showed a significant increase in the expression of multiple ECM genes, including type I collagen (*Col1a1*; significant increase at 2 hours compared to unloaded controls; fold change = 1.6 ± 0.18), lubricin and aggrecan (*Prg4* and *Acan* respectively; both significantly increased at 2 hours and 24 hours compared to unloaded controls; fold change (*Prg4*) = 1.8 ± 0.16, fold change (*Acan*) = 1.6 ± 0.13) (Figure 6A); no significant differences were detected in the expression of type X collagen (*Col10a1*), biglycan (*Bgn*), or decorin (*Dcn*) (Figure S1). We next assessed the expression of genes associated with matrix remodeling. Although no significant differences were detected in the expression of a disintegrin and metalloproteinase with thrombospondin type 1 motif 4 (*Adamts4*) or tissue inhibitor of metallopeptidase 1 (*Timp1*), mRNA levels of matrix metallopeptidase 3 (*Mmp3*) were increased in AF cells following acute exposure to CTS at 0.1 Hz (significantly increased at 2 hours compared to unloaded controls; fold change = 1.6 ± 0.16) (Figure 6B). Acute exposure of AF cells to CTS at 0.1 Hz did not alter inflammatory cytokine gene expression (tumor necrosis factor alpha, *Tnfα*; interleukin 1 beta, *Il‐1β*; interleukin 6, *Il‐6*) or candidate mechanosensitive gene expression (cytochrome c oxidase subunit 2, *Cox2*; MYC proto‐oncogene, *Myc*; Fos proto‐oncogene AP‐1 transcription factor subunit, *Fos*) compared to unloaded controls (Figure 6C, D).

**FIGURE 7:**
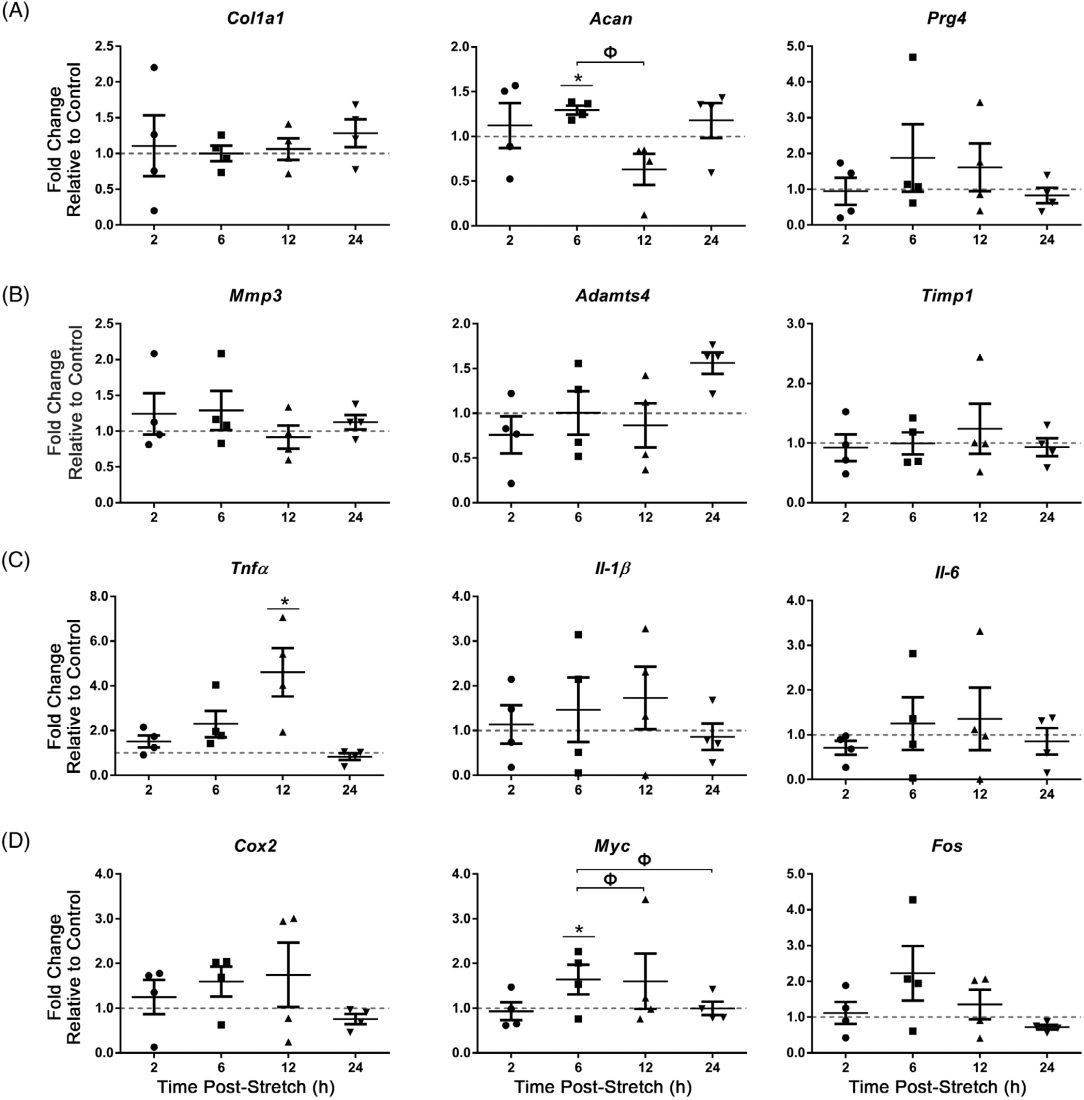
Real‐time PCR analysis of gene expression in annulus fibrosus (AF) cells following acute exposure to CTS at 1.0 Hz. Primary AF cells were subjected to 6% CTS at 1.0 Hz for 30 minutes and RNA harvested following 2, 6, 12 or 24 hours to assess the expression of extracellular matrix genes, A, matrix remodeling genes, B, inflammatory cytokines,C, and candidate mechanosensitive genes, D. AF cells showed a significant increase in the expression of *Acan* (6 hours post‐loading), *Tnfα* (12 hours postloading) and *Myc* (6 hours postloading). Relative gene expression was calculated using ∆∆Ct, normalized for input using the housekeeping gene *Hprt* and expressed relative to time‐matched unloaded controls within each trial (control = 1; indicated as gray dotted lines). Data presented in mean ± SEM; n = 4 cell preparations. Data were analyzed using one‐way analysis of variation (ANOVA) followed by either Dunnet's or Tukey's post hoc test. Grubb's outlier test used to identify outliers. *P < .05 vs unloaded control; Ф = *P* < .05 between fold changes at two time points

AF cells were next subjected to acute (30 minutes) 6% CTS at 1.0 Hz. Compared to CTS at 0.1 Hz, AF cells showed fewer changes in the expression of ECM genes, with significant increases detected in the expression of *Acan* (6 hours postloading; fold change = 1.3 ± 0.05; Figure 7A) and no significant changes in the expression of matrix remodeling genes compared to unloaded controls. No significant differences were detected in the expression of *Col10a1*, *Bgn*, or *Dcn* (Figure S1). Interestingly, CTS at 1.0 Hz induced a significant increase in the expression of *Tnfα* (12 hours post‐loading; fold change = 4.36 ± 1.15; Figure 7C) in AF cells compared to unloaded controls. Among the candidate mechanosensitive genes, 30 minutes CTS at 1.0 Hz induced a significant increase in *Myc* expression (6 hours post‐loading; fold change = 1.6 ± 0.21) in AF cells compared to unloaded controls (Figure 7D).

**FIGURE 8:**
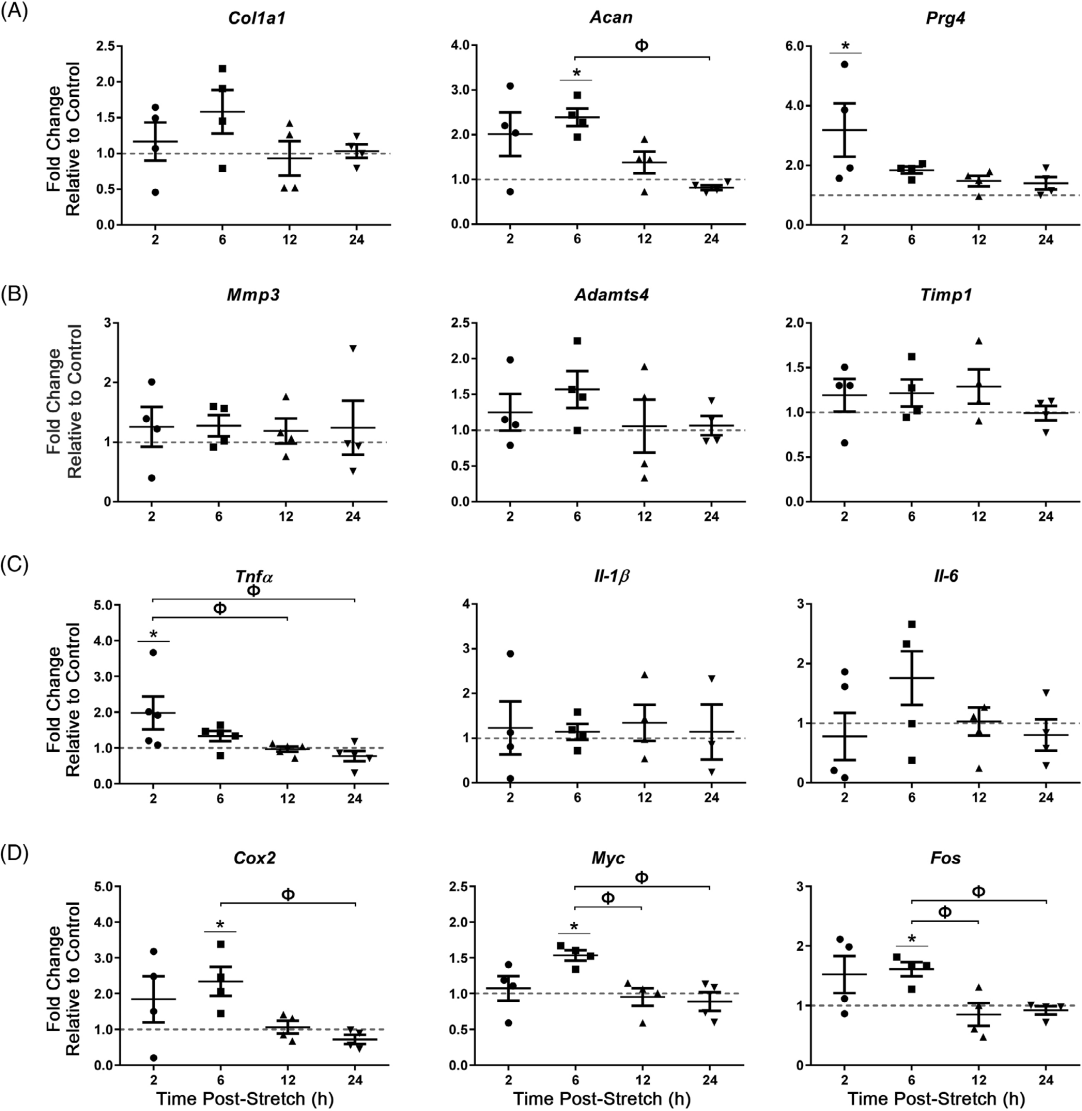
Real‐time PCR analysis of gene expression in annulus fibrosus (AF) cells following acute exposure to CTS at 2.0 Hz. Primary AF cells were subjected to 6% CTS at 2.0 Hz for 30 minutes and RNA harvested following 2, 6, 12 or 24 hours to assess the expression of extracellular matrix genes, A, matrix remodeling genes, B, inflammatory cytokines, C, and candidate mechanosensitive genes, D. AF cells showed a significant increase in the expression of *Acan* (6 hours postloading), *Prg4* (2 hours postloading), *Tnfα* (2 hours postloading), *Cox2* (6 hours postloading), *Myc* (6 hours postloading) and *Fos* (6 hours postloading). Relative gene expression was calculated using ∆∆Ct, normalized for input using the housekeeping gene *Hprt* and expressed relative to time‐matched unloaded controls within each trial (control = 1; indicated as gray dotted lines). Data presented in mean ± SEM; n = 4 cell preparations. Data were analyzed using one‐way analysis of variation (ANOVA) followed by either Dunnett's or Tukey's post hoc test. Grubb's outlier test used to identify outliers. *P < .05 vs unloaded control; Ф = *P* < .05 between fold changes at two time points

Lastly, AF cells were exposed to acute (30 minutes) 6% CTS at 2.0 Hz showed a significant increase in the expression of the ECM genes *Acan* and *Prg4* (6 hours and 2 hours postloading, respectively; fold change (*Acan*) = 2.4 ± 0.12, fold change (*Prg4*) = 3.2 ± 0.89) compared to unloaded controls (Figure 8A). No significant differences were detected in the expression of *Col10a1*, *Bgn*, or *Dcn* (Figure S1) or genes associated with matrix remodeling (Figure 8B). Acute CTS at 2.0 Hz induced a significant increase in *Tnfα* gene expression (2 hours postloading; fold change = 2.2 ± 0.52; Figure 8C) in AF cells compared to unloaded controls. Notably, all candidate mechanosensitive genes assessed (*Cox2*, *Myc*, *Fos*) were significantly upregulated compared to the unloaded controls at 6 hours following exposure of AF cells to acute CTS at 2.0 Hz (fold change (*Cox2*) = 2.3 ± 0.45, fold change (*Myc*) = 1.5 ± 0.07, fold change (*Fos*) = 1.6 ± 0.13; Figure 8D).

**FIGURE 9:**
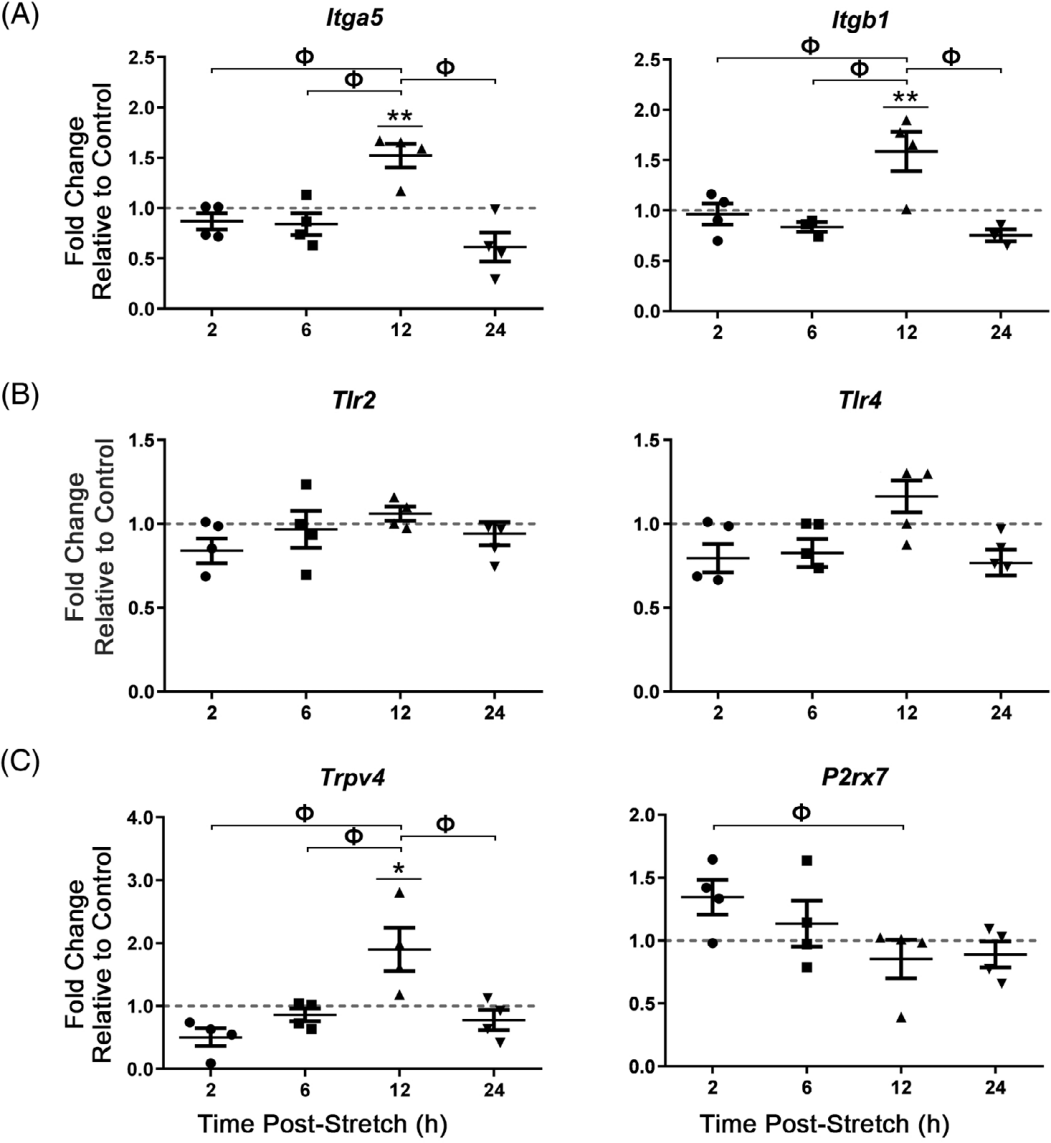
Real‐time PCR analysis of candidate cell surface receptor gene expression in annulus fibrosus (AF) cells following acute exposure to CTS at 2.0 Hz. Primary AF cells were subjected to 6% CTS at 2.0 Hz for 30 minutes and RNA harvested following 2, 6, 12 or 24 hours to assess the expression of integrin subunits, A, Toll‐like receptors, B, and membrane ion channel receptors, C. AF cells showed a significant increase in the expression of *Itgα5* (12 hours postloading), *Itgβ1* (12 hours postloading), and *Trpv4* (12 hours postloading). Relative gene expression was calculated using ∆∆Ct, normalized for input using the housekeeping gene *Hprt* and expressed relative to time‐matched unloaded controls within each trial (control = 1; indicated as gray dotted lines). Data presented in mean ± SEM; n = 4 cell preparations. Data were analyzed using one‐way analysis of variation (ANOVA) followed by either Dunnett;s or Tukey's post hoc test. Grubb's outlier test used to identify outliers. *P < .05 vs unloaded control; **P < .01 vs unloaded control; Ф = *P* < .05 between fold changes at two time points

Given that CTS at 2.0 Hz elicited the most robust changes in the expression of ECM, pro‐inflammatory cytokine, and mechanosensitive genes in AF cells compared to other loading protocols, we assessed if the expression of cell surface receptors would likewise be altered by these parameters of mechanical load. AF cells exposed to CTS at 2.0 Hz showed a significant increase in the gene expression of integrin subunits, *Itgα5* (12 hours postloading; fold change = 1.4 ± 0.12) and *Itgβ1* (12 hours postloading; fold change = 1.6 ± 0.13) compared to unloaded controls (Figure 9A). Acute (30 minutes) exposure of AF cells to CTS at 2.0 Hz did not significantly alter the expression of both toll‐like receptor 2 (*Tlr2*) and toll‐like receptor 4 (*Tlr4*) genes (Figure 9B). In contrast, acute exposure of AF cells to CTS at 2.0 Hz significantly upregulated the expression of the transient receptor potential vanilloid 4 (*Trpv4*) gene at 12 hours postloading (fold change = 1.9 ± 0.34), with a trend towards increased purinoreceptor x subtype 7 (*P2rx7*) gene expression 2 hours postloading (fold change = 1.3 ± 0.14), compared to unloaded controls (Figure 9C).

All of the gene expression data from three different CTS protocols were compared at each time points to test whether changes in gene expression is frequency dependent. The analysis identified frequency as a significant source of variation in the expression of *Acan*, *Myc*, and *Tnfα* genes (Figure S2).

## 4 DISCUSSION

At the cellular level, mechanical loading in the IVD regulates a variety of biological processes in both cell type‐ and stimulus‐dependent manner. Considering the dynamic nature of the loading environment in the IVD, previous studies examined the effects of CTS on AF cells, focusing on changes in the expression of genes related to ECM anabolism and catabolism.^28^, ^29^, ^30^, ^31^, ^32^ Loading frequency, among other parameters of CTS, has been shown to be an important factor in determining cellular response to mechanical load.^56^ Building upon these findings, the present study aimed to characterize the mechano‐response of primary murine AF cells grown in a dynamic culture system that delivers bi‐axial CTS. Using this model, we showed that AF cells adapted to different load frequencies by remodeling their cytoskeletal microarchitecture and activating intracellular signaling pathways dependent on the frequency of the applied load. Furthermore, we demonstrated that varying loading frequencies altered the mechano‐response of AF cells at the level of gene expression, with notable changes in the expression of extracellular matrix, pro‐inflammatory cytokines, mechano‐sensitive genes, and cell surface receptors. Taken together, these data provide further evidence for the existence of a “window” of loading parameters that may be beneficial to the IVD health.

The design of the present study incorporated two essential validation steps. First, we incorporated both IVD‐specific genetic labeling strategies and gene expression analysis to assess and validate the AF cell phenotypes in our in vitro culture system. This analysis enabled us to confirm that primary cell preparations did not contain NP cells and also enabled us to assess the effects of the culture substrate on cell phenotype, since fibroblasts have been shown to exhibit matrix stiffness adaptation.^57^ Gene expression analysis demonstrated that on both tissue culture plastic and deformable silicone membranes, primary AF cells maintained robust expression of the AF‐associated markers *Col1a1*,^44^ *Gdf10*,^45^ the marker of sclerotome development, *Pax1*,^48^ and *Cilp* ^49^ a gene recently reported by our group as enriched in the murine AF compared to the NP.^50^ In addition, we assessed the expression of *Acta2* and *Fap*, two markers of the fibroblast phenotype.^51^, ^52^ Primary AF cells showed increased expression of *Acta2* and similar expression of *Fap* compared to intact AF tissues. Alpha smooth muscle actin (α‐SMA), protein encoded by *Acta2*, is a contractile actin isoform found in myofibroblast.^51^ In the context of the IVD, previous studies showed α‐SMA expression in AF cells in both the canine and human IVD^62^, ^63^ as well as primary AF cells in 2D culture.^62^ Buckley et al^64^ and Desmouliere et al^65^ independently showed that α‐SMA plays a role in cell attachment, maintenance of cell shape, and may contribute to production and accumulation of ECM. *Fap* is a specific marker of the activated fibroblast phenotype and implicated in pathological tissue fibrosis^52^; no differences were detected in the current study between the expression of *Fap* in primary AF cells compared to intact AF tissues. These data confirm that AF cells maintain their phenotype with increased expression of microarchitecture proteins for attachment and matrix production. Second, we confirmed the peak strain and strain uniformity of the MCB1 device by motion tracking analysis and showed that the CTS was being sensed by the AF cells by assessing stress fiber formation. Bioreactor systems described in previous studies were associated with limitations, including nonuniform strain patterns and heterogeneity in type of loading delivered (ie, biaxial at central area, but approached pure uniaxial strain toward the edge).^66^, ^67^, ^70^ Our validation demonstrated that the MCB1 device overcame these limitations by incorporating a design mechanism that transfers linear motion into 24 points of radial stretch, which resulted in delivery of uniform biaxial strain on the silicone membrane.

One of the earliest responses we detected in AF cells exposed to CTS was transient activation of ERK1/2, but this was only observed at our highest frequency loading (6% CTS at 2.0 Hz). MAPKs are part of a phospho‐relay signaling pathway known to mediate stress responses in different cell types, including IVD cells,^33^, ^34^, ^35^, ^36^, ^37^, ^38^ making them potential mediators of mechanotransduction. Previous study by Pratsinis et al showed that in human AF cells derived from degenerate IVDs, 4% uniaxial CTS at 1.0 Hz induced the activation of all three MAPK pathways (ERK. P38, JNK) immediately after CTS, independent of strain magnitude of the applied load.^38^ In contrast, the present study detected only significant activation of ERK1/2 in healthy murine AF cells. These differences may be due to the different species of origin of the AF cells used, differences in loading parameters, or differences associated with isolating cells from healthy or degenerate tissues. Functionally, integrin‐dependent activation of ERK1/2, Src and RhoA has been found to regulate stress fiber formation in other cell types, including endothelial cells and osteocytes.^71^, ^72^, ^73^ Given that increased stress fiber formation in AF cells was induced by increasing frequency of CTS in our study, early activation of ERK1/2 may be required for cytoskeletal rearrangement in AF cells. Of note, a study by Hirata et al showed that phosphorylated ERK1/2 proteins localize to the stress fibers upon mechanical stretch, suggesting that stress fibers serve as a platform for tension‐induced activation of biochemical mechanotransduction pathways.^74^ As such, the link between cytoskeletal tension, stress fiber formation and the role of ERK1/2 activation should be further explored in AF cells.

Our findings are similar to previous studies reporting an anabolic response of matrix gene expression in AF cells subjected to CTS.^28^, ^29^, ^31^, ^32^, ^58^ Interestingly, our study demonstrated that exposure of AF cells to CTS at low (0.1 Hz) and high (2.0 Hz) frequencies induced a significant increase in the gene expression of *Prg4*. Lubricin, the protein encoded by *Prg4*, is a large mucinous glycoprotein that serves as the primary boundary lubricant for articular cartilage^75^ and its expression has been found to be protective against the development of osteoarthritis.^76^ Previous studies reported lubricin expression in the interlamellar space of the annular lamellae in caprine IVDs.^77^ Our data suggest that similar to chondrocytes in which *Prg4* expression is regulated by shear force,^78^ *Prg4* is mechanically regulated in the AF.

We also assessed the effects of CTS‐exposure on AF cell expression of candidate mechano‐sensitive genes identified in related musculoskeletal cell types. Previous studies reported that in osteoblasts, expression of *Cox2*, *Myc*, and *Fos* genes are induced by various modes of mechanical stimulation (vibration, fluid shear) and muscle cells also increase *Fos* gene expression following exposure to 20% cyclic tensile strain.^53^, ^54^, ^55^ The most robust and consistent changes were observed in AF cells exposed to CTS 2.0 Hz, which induced a significant upregulation of *Cox2*, *Myc*, and *Fos* gene expression. Given that expression of all three of these genes is required for transition from G1 to S phase of the cell cycle,^55^, ^56^, ^57^ these findings suggest that AF cells exposure to higher frequency CTS may promote cell proliferation. A previous study demonstrated that expression of a dominant negative form of Rho GTPase in osteoblasts blocked fluid shear stress‐induced stress fiber formation and the expression of the immediate early genes *Cox2* and *Fos*.^57^ Future studies using this model system will build on the characterization of the acute effects of CTS, and specifically assess the role of Rho GTPase and the changes in cell proliferation in AF cells following chronic exposure to CTS.

The pathophysiology of IVD degeneration is associated with alterations to the ECM due to imbalances between synthesis and degradation of ECM proteins. It has been hypothesized that ECM degradation fragments induce local inflammation driven by cells of the IVD.^79^ However, the role of mechanical stimulation in either the initiation or propagation of local inflammation is largely unknown. Previous studies report that IVD cells exposed to high mechanical strain at low frequency (20% at 0.001 Hz) show increased gene expression of inflammatory receptors and cytokines.^30^ In keeping with these findings, the present study showed that exposure of AF cells to CTS at 1.0 Hz and 2.0 Hz induced a significant upregulation of *Tnfα* gene expression, suggesting that mechanical stimulation alone can directly contribute to the initiation of local inflammation. We acknowledge, however, that these findings are limited to quantification of changes at the level of gene expression; more long‐term experiments should explore how alterations in gene expression alter levels of secreted inflammatory cytokines. Although traditionally inflammation was viewed as detrimental response involved in disease progression, recent reports suggest that a balanced inflammatory response may be required for matrix repair and maintaining tissue homeostasis in different cell types, including IVD cells.^79^, ^80^ Given that the loading parameters used in this study fall within the range of those experienced in a physiological context, the increased expression of *Tnfα* may be contributing to matrix homeostasis. Nonetheless, the data suggest a direct link between mechanical stimulation and inflammatory cytokine gene expression in AF cells.

At the cellular level, mechanical signals are transduced via cell surface receptors acting in concert to translate physical force into biologically relevant signals.^32^, ^72^ Previous studies using dynamic organ culture showed that expression of integrin subunits α5 and β1 genes was induced in both rat NP and AF tissues by cyclic compressive stress (1.3 MPa, 1.0 Hz) for 6 days.^81^ Le Maitre et al^82^ showed that compression‐induced changes in matrix gene expression in human NP cells were mediated by α5β1 integrin signaling. Our findings of mechanically induced *Itgα5* and *Itgβ1* expression suggest that α5β1 integrin signaling may likewise contribute to AF mechanotransduction in response to CTS, in keeping with the downstream activation of the ERK1/2 pathway we report. Gawri et al^30^ showed increased expression of both the toll‐like receptor 2 and 4 genes in human IVD cells exposed to high mechanical strain at low frequency (20% cyclical stretch at 0.001 Hz). The observed difference from the current study, which showed no change in TLR expression in murine AF cells may be due to differences in loading parameters, such as strain percentage, frequency, and duration. Lastly, *Trpv4* and *P2rx7*, candidate mechano‐sensitive channels studied in other musculoskeletal cell types^58^, ^59^, ^60^ were upregulated in AF cells following exposure to CTS. Previous studies reported that the expression of both genes was regulated by cyclic compression in chondrocytes.^58^, ^59^ Functionally, both TRPV4 and P2X7 have been shown to elicit intracellular calcium transient upon mechanical compression.^58^, ^59^ In the context of the IVD, TRPV4 is activated by changes in osmolarity,^83^ and a related purinoreceptor, P2X4, has been shown to mediate ATP‐induced membrane potential response.^84^ Taken together, the current study demonstrates that AF cells adapt to mechanical stimulation by regulating cell surface receptor gene expression, thereby potentially modulating the activation of intracellular signaling pathways.

A limitation of the current study is the examination of cellular responses to CTS in a cell culture system where cells were seeded onto silicone membranes; consequently, endogenous cell‐matrix interactions present in the IVD microenvironment were not recapitulated. In order to minimize these issues, AF cells were precultured on membranes in media supplemented with ascorbic acid to enable collagen production and secretion. To more fully explore the effect of ECM in mediating mechanical strain, future studies could incorporate specific ECM protein coatings in the MCB1 device. We also acknowledge that our protocols for primary murine cell isolation may have introduced heterogeneity in the AF cell population studied. To maximize the yield of our primary cell isolation from the murine IVDs, we pooled AF tissues from all anatomical regions (cervical through caudal) and included cells from both inner and outer AF. Subtle differences in the phenotype of AF cells in each of these regions, or in the mechanical loading environment from which they were isolated may impact their response to mechanical stimulation in vitro.

## 5 CONCLUSIONS

Overall, our findings suggest that effects of CTS on healthy murine AF cells are frequency dependent. The most anabolic effect marked by increased expression of extracellular matrix genes was observed following exposure to 6% CTS at 0.1 Hz. At higher frequency (2.0 Hz), CTS induced mechanically sensitive genes (associated with the regulation of cell cycle progression) and proinflammatory cytokine gene expression in healthy AF cells. Taken together, these finding further suggests the existence of a range of loading parameters that are beneficial to IVD health, which may help to understand the pathophysiology of IVD degeneration in the context of mechanobiology. Moreover, the differences in MAPK activation we report in AF cells derived from healthy IVDs compared to previous findings from degenerate tissues suggest differences in the cellular signaling pathways activated by CTS in AF cells associated with tissue health. Further studies are needed to elucidate other mechanotransduction pathways and their involvement in regulating cellular function in the IVD.

## CONFLICT OF INTEREST

The authors declare that they have no competing interests.

## AUTHORS CONTRIBUTION

All authors were involved in drafting the article or revising it critically for content, and all authors approved the final version. Dr. Séguin had access to all of the data in the study and takes responsibility for the integrity of the data and the accuracy of its analysis. Min Kyu M. Kim and Cheryle A. Séguin: study conception and design; Min Kyu M. Kim, Marissa J. Burns, and Meaghan E. Serjeant: acquisition of data; Min Kyu M. Kim, Marissa J. Burns, Meaghan E. Serjeant, and Cheryle A. Séguin: analysis and interpretation of data.

## Supporting information


**Supplemental Figure S1** **Effects of CTS on the expression of additional candidate ECM genes in AF cells.** Biglycan (*Bgn*), type X collagen (*Col10a1*), and decorin (*Dcn*) gene expression was quantified in AF cells exposed to acute CTS at 0.1 Hz (A), 1.0 Hz (B), and 2.0 Hz (C). The expression levels of the three matrix genes did not change upon mechanical stimulation. Relative gene expression was calculated using the ∆∆Ct method, normalized for input using the housekeeping gene *Hprt* and expressed relative to time‐matched unloaded controls within each trial (control = 1; indicated as gray dotted lines). Data presented in mean ± SEM; n = 4 cell preparations. Data were analyzed using one‐way ANOVA followed by either Dunnett;s or Tukey's post‐hoc test. Grubb's outlier test used to identify outliers. Ф = *P* < 0.05 between fold changes at two time points.

**Supplemental Figure S2** **Two way ANOVA table with P and F value for each gene.** Changes in the expression levels of genes outlined above at each loading protocol were compared using two‐way ANOVA followed by Tukey's post‐hoc test. For a given gene, the table outlines the F and p values for the two sources of variation (variables).
