# Supplementary figures and images for: The mechano‐response of murine annulus fibrosus cells to cyclic tensile strain is frequency dependent
Source: JOR Spine. 2020 Jul 20;3(4):e21114. doi: 10.1002/jsp2.1114 (PMC7770207; doi:10.1002/jsp2.1114)

(A)

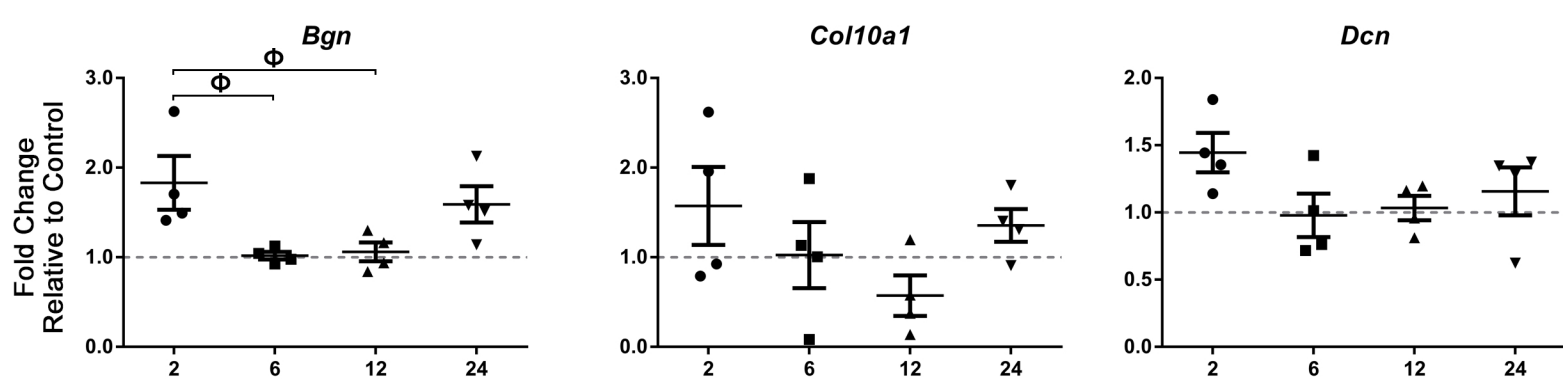

(B)

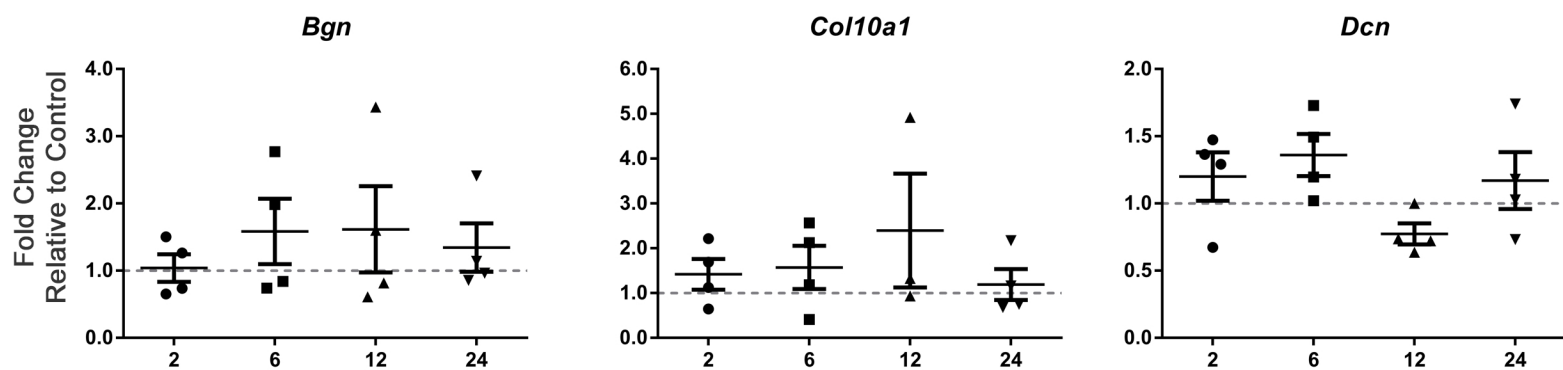

(C)

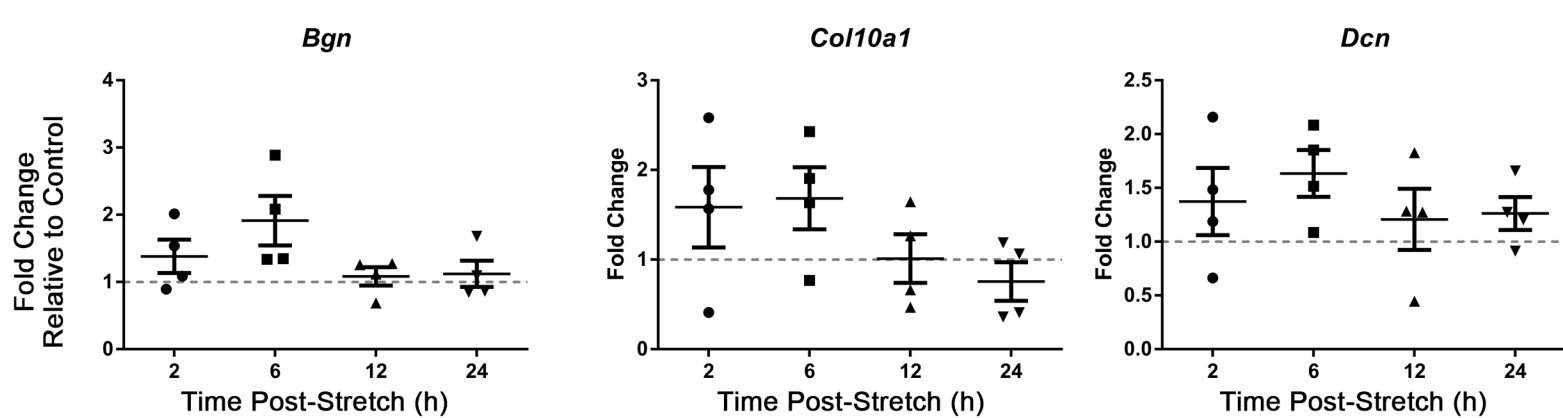

Supplement: Supplementary file 1 — Supplemental Figure S1 Effects of CTS on the expression of additional candidate ECM genes in AF cells. Biglycan (Bgn), type X collagen (Col10a1), and decorin (Dcn) gene expression was quantified in AF cells exposed to acute CTS at 0.1 Hz (A), 1.0 Hz (B), and 2.0 Hz (C). The expression levels of the three matrix genes did not change upon mechanical stimulation. Relative gene expression was calculated using the ∆∆Ct method, normalized for input using the housekeeping gene Hprt and expressed relative to time‐matched unloaded controls within each trial (control = 1; indicated as gray dotted lines). Data presented in mean ± SEM; n = 4 cell preparations. Data were analyzed using one‐way ANOVA followed by either Dunnett;s or Tukey's post‐hoc test. Grubb's outlier test used to identify outliers. Ф = P < 0.05 between fold changes at two time points. [file JSP2-3-e21114-s001.pdf]
