# Supplementary material for: The mechano‐response of murine annulus fibrosus cells to cyclic tensile strain is frequency dependent
Source: JOR Spine. 2020 Jul 20;3(4):e21114. doi: 10.1002/jsp2.1114 (PMC7770207; doi:10.1002/jsp2.1114)
Supplement: Supplementary file 2 — Supplemental Figure S2 Two way ANOVA table with P and F value for each gene. Changes in the expression levels of genes outlined above at each loading protocol were compared using two‐way ANOVA followed by Tukey's post‐hoc test. For a given gene, the table outlines the F and p values for the two sources of variation (variables). [file JSP2-3-e21114-s002.docx]

| Gene | Source of Variation | F | p value | Significant? |
| --- | --- | --- | --- | --- |
| *Acan* | Time | 8.505 | <0.0001 | Yes |
|  | Frequency | 4.189 | 0.0215 | Yes |
| *Adamts4* | Time | 1.392 | 0.2519 | No |
|  | Frequency | 1.425 | 0.2511 | No |
| *Col1a1* | Time | 2.982 | 0.0288 | Yes |
|  | Frequency | 0.5803 | 0.5639 | No |
| *Cox2* | Time | 4.237 | 0.0054 | Yes |
|  | Frequency | 0.3675 | 0.6946 | No |
| *Fos* | Time | 4.017 | 0.0072 | Yes |
|  | Frequency | 0.2096 | 0.8117 | No |
| *Il-1β* | Time | 0.4179 | 0.7948 | No |
|  | Frequency | 0.2114 | 0.8102 | No |
| *Il-6* | Time | 0.3325 | 0.8546 | No |
|  | Frequency | 0.03311 | 0.9675 | No |
| *Mmp3* | Time | 1.147 | 0.3469 | No |
|  | Frequency | 1.017 | 0.3697 | No |
| *Myc* | Time | 4.409 | 0.0043 | Yes |
|  | Frequency | 3.506 | 0.0385 | Yes |
| *Prg4* | Time | 2.456 | 0.0592 | No |
|  | Frequency | 2.622 | 0.0838 | No |
| *Timp1* | Time | 0.7253 | 0.5793 | No |
|  | Frequency | 0.9733 | 0.3856 | No |
| *Tnfα* | Time | 3.137 | 0.0233 | Yes |
|  | Frequency | 3.811 | 0.0296 | Yes |
